# Human Genes Encoding Transcription Factors and Chromatin-Modifying Proteins Have Low Levels of Promoter Polymorphism: A Study of 1000 Genomes Project Data

**DOI:** 10.1155/2015/260159

**Published:** 2015-08-31

**Authors:** Elena V. Ignatieva, Victor G. Levitsky, Nikolay A. Kolchanov

**Affiliations:** ^1^Laboratory of Evolutionary Bioinformatics and Theoretical Genetics, Federal State Research Center Institute of Cytology and Genetics, Russian Academy of Sciences, Siberian Branch, Novosibirsk 630090, Russia; ^2^Department of Natural Science, Novosibirsk State University, Novosibirsk 630090, Russia; ^3^Laboratory of Bioinformatics, Federal State Research Center Institute of Cytology and Genetics, Russian Academy of Sciences, Siberian Branch, Novosibirsk 630090, Russia; ^4^Center for Brain Neurobiology and Neurogenetics, Federal State Research Center Institute of Cytology and Genetics, Russian Academy of Sciences, Siberian Branch, Novosibirsk 630090, Russia; ^5^Systems Biology Department, Federal State Research Center Institute of Cytology and Genetics, Russian Academy of Sciences, Siberian Branch, Novosibirsk 630090, Russia

## Abstract

The expression level of each gene is controlled by its regulatory regions, which determine the precise regulation in a tissue-specific manner, according to the developmental stage of the body and the necessity of a response to external stimuli. Nucleotide substitutions in regulatory gene regions may modify the affinity of transcription factors to their specific DNA binding sites, affecting the transcription rates of genes. In our previous research, we found that genes controlling the sensory perception of smell and genes involved in antigen processing and presentation were overrepresented significantly among genes with high SNP contents in their promoter regions. The goal of our study was to reveal functional features of human genes containing extremely small numbers of SNPs in promoter regions. Two functional groups were found to be overrepresented among genes whose promoters did not contain SNPs: (1) genes involved in gene-specific transcription and (2) genes controlling chromatin organization. We revealed that the 5′-regulatory regions of genes encoding transcription factors and chromatin-modifying proteins were characterized by reduced genetic variability. One important exception from this rule refers to genes encoding transcription factors with zinc-coordinating DNA-binding domains (DBDs), which underwent extensive expansion in vertebrates, particularly, in primate evolution. Hence, we obtained new evidence for evolutionary forces shaping variability in 5′-regulatory regions of genes.

## 1. Introduction

The expression of eukaryotic protein-coding genes can be regulated at several steps, including transcription initiation and elongation, mRNA processing and transport, translation, and stability. Most of the regulatory processes, however, are believed to occur at the level of transcription initiation [1].

Transcription is precisely regulated depending on cellular conditions. The transcriptional activity of each gene is regulated by its promoter region, which is typically located upstream and immediately adjacent to the transcription start site (TSS). Promoters contain specific short regions of DNA (10–20 nucleotides) recognized by regulatory proteins (transcription factors) and termed transcription factor-binding sites. Specific interaction of transcription factors with DNA sequences within the promoter region (alone or in an assemblage with other proteins) facilitates the recruitment of RNA polymerase to specific genes [1, 2].

Regulatory regions of eukaryotic genes are typically organized in a complicated manner, so that the regulatory regions of a specific gene may contain binding sites for more than 20 transcription factors [3–6]. On the other hand, a great number of different regulatory proteins (general transcription factors, regulatory sequence-specific DNA-binding factors, transcriptional coregulators, etc.) are involved in transcription regulation. According to recent data, the human genome encodes about 1500 regulatory sequence-specific DNA-binding factors (transcription factors, TFs) [7–9].

TFs constitute a large functional family of proteins directly regulating the activity of genes. To exert their function in gene transcription activation or repression, TFs must recognize the place in the genome where they should bind. For this purpose, they are equipped with DNA-binding domains (DBDs) [8].

Another very important group of regulatory proteins affecting transcription are chromatin regulators. Chromatin regulators can mediate histone (or DNA) modifications and chromatin remodeling to adjust chromatin structures and functions [1]. The inspection of databases, comprising human genes involved in chromatin regulation (CREMOFAC, CR Cistrome, and HIstome), and annotation by GO terms associated with chromatin, presented by EntrezGene, tells us that at least one hundred chromatin regulators may be encoded by the human genome [10–12].

A single nucleotide polymorphism, or SNP, is a variation at a single position in a DNA sequence among individuals. The 1000 Genomes Project characterizes human genomic variation by using next-generation sequencing strategies. At present, the project reports on genomes of 1092 individuals sampled from 14 populations drawn from Europe, East Asia, sub-Saharan Africa, and the Americas. Over 38 million SNPs have been identified by the 1000 Genomes Project, more than a half of which were not described previously [13].

There is evidence that promoter regions are particularly stressed by transcription-related mutagenic phenomena and that they harbor a large amount of genetic variations compared with other genomic regions. According to our previous study [14], which was based on NCBI's dbSNP build 138, more than half of the total number of SNPs (59.05%) identified by the 1000 Genomes Project are located in transcribed regions of the human genome, 1.07% of all SNPs are mapped to coding exons, and 1.05% are located within promoter regions of genes. The SNP density in the 500 bp regions upstream of TSSs is approximately the same as in introns (3.7 SNPs per 1000 bp). It is considerably higher than in coding regions (2.4 SNPs per 1000 bp).

As well as SNPs located in coding gene regions, promoter and enhancer SNPs may affect phenotypic traits. One functional mechanism is that the genetic variants within upstream regions may influence gene transcription by altering the binding affinity of a transcription factor to the DNA [1, 15–17]. Such SNPs are designated as regulatory.

For example, it was estimated that the G → T substitution (rs1271572) in the* ERβ* promoter prevented transcription factor Yin Yang 1 (YY1) binding and reduced its transcription activity. The TT genotype for rs1271572 was associated with elevated risk for breast cancer in Chinese women and with unfavorable prognosis in Chinese breast cancer patients [18].

At present, evidence for evolutionary and nonevolutionary forces shaping the genetic variability of 5′-flanking regions of human genes is under investigation [19]. In this context, functional analysis of genes whose promoters harbor extremely high or very low SNP contents may be useful.

In our previous study based on the human whole-genome data from the 1000 Genomes Project, functional analysis of genes whose 5′-flanking regions contain high SNP contents (six or more SNPs) was performed. We revealed two overrepresented groups: (1) genes controlling the sensory perception of smell and (2) genes involved in antigen processing and presentation [14]. We suggested that high promoter SNP contents caused diversity in the expression levels of genes and, in turn, were partly responsible for the broad variability of immune recognition and olfactory cognition. We conjectured that the parallelism between functions of the immune and olfactory systems was due to the fact that both systems were targeted on the reception of extremely variable chemical compounds (numerous environmental olfactory stimuli or immune stimuli produced by rapidly evolving microbiota). Therefore, high SNPs contents in the promoters of genes involved in olfactory cognition and antigen processing and presentation may be, to some extent, a result of balancing selection.

Functional analysis of genes with high SNP content in regulatory regions was performed by the FANTOM5 Consortium. They found that SNPs associated with such diseases as Hodgkin's lymphoma, inflammatory bowel disease (early onset), systemic sclerosis (and the like), and such phenotypic traits as birth weight and prostate-specific antigen levels (and the like) are significantly overrepresented in regulatory regions (promoter and enhancers) [20].

The goal of this study is to reveal functional characteristics of human genes containing extremely low level of SNPs in promoter regions. This knowledge may give a deeper view of genic intolerance to regulatory variation and may be useful for interpretation of personal genomes. Investigating data from the 1000 Genomes Project Consortium, we found that almost one-fifth (16.5%) of the total number of transcripts did not contain SNPs in their 500 bp long upstream regions. Functional analysis of transcripts (genes) with SNP-depleted 5′-regulatory regions revealed several overrepresented functional groups of genes controlling: (1) gene-specific transcription, (2) chromatin organization, and (3) male gamete generation.

Then comparisons among all genes encoding transcription factors (or chromatin-modifying proteins or four superclasses of TFs) versus genes from the whole-genome were done. Analysis of transcript distributions as a function of SNP contents per 700 bp regions (−600/+100) around TSSs showed that SNP contents in the main groups of genes/transcripts (TFs and chromatin-modifying proteins) and in three superclasses of TFs (with the exception of TFs with zinc-coordinating DBDs) were lower than the SNP content in the whole-genome set of transcripts. In addition, a similar analysis was performed for genes located on autosomes (chromosomes 1–22) and reduced genetic variability of upstream regions controlling transcription factors and chromatin-modifying proteins was observed in these cases as well.

Finally, the main functional gene groups (TFs and chromatin-modifying proteins) and genes belonging to four different superclasses of TFs were ranked according to their RVIS values, calculated from exome data by Petrovski et al. [21]. The differences between groups of genes revealed by using RVIS values were in good agreement with the differences between these gene groups and the whole-genome dataset revealed when distributions of promoter SNPs content were analyzed. As RVIS correlates with the functional significance of genes, we conclude that the reduced level of SNPs in the 5′-regulatory regions of genes encoding TFs and chromatin-modifying proteins may be explained, at least in part, by genic intolerance to regulatory variation.

## 2. Materials and Methods

### 2.1. Sequence Sets and SNP Data

The annotations of SNPs mapped to chromosomes 1–22, X, and Y of the GRCh37/hg19 assembly of the human genome were extracted from the UCSC Table Browser (https://genome.ucsc.edu/cgi-bin/hgTables, the track* common SNPs (142)*, and table* snp142Common*; this track refers to release142 of dbSNP, http://www.ncbi.nlm.nih.gov/projects/SNP/). For SNP data, we used additional filters* class single* and* validation by 1000 genomes*.

The annotations of transcripts for the GRCh37 assembly of the human genome were extracted from the Ensembl archive by the Biomart data mining tool (http://grch37.ensembl.org/biomart/martview). The following criteria were used to retrieve annotations of transcripts: (a) with HGNC ID(s): only; (b) gene type: protein_coding; (c) transcript type: protein_coding; (d) chromosome: 1–22,X,Y; (e) status (gene): KNOWN; (f) status (transcript): KNOWN. As a result, the total whole-genome set of 47,469 transcripts with distinct transcription start sites (TSSs) was obtained. These transcripts were annotated by 18,817 distinct HGNC gene symbols (see Table 1, dataset* whole-genome*).

At the first step, the SNP content was determined for each transcript as the count of SNPs in the 500 bp long region upstream of the annotated TSS.

Then we divided regions from −1000 to +200 around each TSS into bins of 100 bp. For each transcript, the count of SNPs in each bin was determined.

Finally, we calculated the counts of SNPs in seven regions with different locations surrounding TSSs ([−900/+100], [−800/+100], etc., until [−300/+100]). In each case, we revealed subsets of transcripts whose 5′-regulatory regions did not contain SNPs. These subsets are designated below as* SNP-depleted.*

### 2.2. GO Category and Pathway Analysis

The Database for Annotation, Visualization, and Integrated Discovery web-based Functional Annotation Tool (DAVID tool) was applied [22, 23] to the sets of SNP-depleted transcripts that do not contain SNPs in their regions surrounding TSSs.

The overrepresented GO terms from the* biological processes* vocabulary were considered in our study. The significance of GO terms was estimated through the EASE score, a modified Fisher exact *p* value (a built-in function of DAVID tool) on the base of the number of genes from the list under study and the number of genes expected by chance. Groups with fold enrichment values 1.5 or more and *p* values (EASE scores) less than 0.001 were kept in analysis.

### 2.3. Functional Groups of Genes

The set of genes encoding TFs (designated below as* All TFs*) was obtained using the TFClass database (http://tfclass.bioinf.med.uni-goettingen.de/) [9]. TFClass provides a comprehensive classification of human transcription factors based on their DBDs. A total of ten superclasses (including the transitory Superclass “0,” “Yet undefined DNA-binding domains”) have been identified, comprising 40 classes and 111 families. Counted by genes, 1558 human TFs have been classified so far. The data (format-version 1.2., date 23:09:2014) were downloaded in OBO format and then processed to the tab-delimited text format. Identifiers from Ensembl database were converted to EntrezGene database identifiers with the bioDBnet: db2db tool (http://biodbnet.abcc.ncifcrf.gov/db/db2db.php).

In addition, four subsets of the set* All TFs* were formed. According to TFClass, Superclass 2 (zinc-coordinating DBDs) was by far the largest among the nine superclasses of defined DBDs. It included 51% of all TF genes, followed by helix-turn-helix (27%) and basic domain factor genes (11%). Therefore, the subsets* ZNF*,* HTH*, and* Basic* comprised genes encoding factors with zinc-coordinating DBDs, helix-turn-helix, and basic domain factor genes, respectively. In addition, the last subset* Other* included genes (11%) encoding factors with DBDs of all other types (the remaining seven superclasses).

The set of human genes encoding chromatin-modifying proteins (this set of transcripts/genes was designated as* Chr_Mod*) was compiled from three sources. First, 99 genes encoding chromatin-modifying proteins were extracted from EntrezGene (http://www.ncbi.nlm.nih.gov/gene) using the GO term “chromatin modification” as a query. Second, 64 genes were obtained from CREMOFAC, a database of chromatin-remodeling factors [10]. Third, 23 genes were picked out from CR Cistrome, a knowledgebase for chromatin-modifying enzymes and chromatin remodelers [11]. CR Cistrome comprised genes encoding chromatin regulators from four cohorts: reader, writer, eraser, and remodeler. After fusion of the three thus obtained gene lists, the resulting gene set comprised 167 genes encoding proteins with chromatin-modifying activities.

The human genes encoding proteins involved in spermatogenesis (this set of genes/transcripts was designated as* Sperm*) were extracted from EntrezGene (http://www.ncbi.nlm.nih.gov/gene) using the GO term “spermatogenesis” as a query.

Table S1, in Supplementary Material available online at http://dx.doi.org/10.1155/2015/260159, presents the lists of transcripts for all groups used in analysis. The numbers of transcripts/genes for all groups are given in Table 1.

### 2.4. Comparison of SNP Content Distributions in 5′-Regulatory Regions of Functional Gene Groups with the Distribution in the Whole-Genome Dataset

Another approach was based on the analysis of the distributions of SNP content in 5′-regulatory regions of human genes from functional gene groups (encoding TFs or chromatin-modifying proteins).

The distributions of SNP contents in 5′-regulatory regions for any test group of transcripts were compared with the distribution for the whole-genome dataset. The statistical significance of differences was estimated by Fischer's *t*-test for angular (arcsine square root) transformed proportions [24]. The first proportion *p*_1_(*N*) was computed for the test group as the ratio of the number of transcripts having no more than *N* SNPs in 5′-regulatory regions to the total number of transcripts in the test group. The second proportion *p*_2_(*N*) was calculated similarly for the whole-genome dataset. For the range of thresholds *N* (0,1, 2,…) the angular transformation *y*(*p*_*i*_) was computed to apply the *t*-test as follows: *y*(*p*_*i*_) = 2arcsin(sqrt(*p*_*i*_)), where *i* = 1,2.

### 2.5. The Estimation of Genic Intolerance to Functional Variation for Genes from Functional Gene Groups and the Comparison with That in the Whole-Genome Dataset

To compare genic intolerance to functional variation for functional groups of genes considered above with that for the whole-genome dataset, we used RVIS (Residual Variation Intolerance Score) values presented in Dataset S2 from [21]. They presented RVISs for 16,956 human genes: when the score was equal to zero, the gene has an average number of common functional variants, given its total mutational burden; when the score was negative, the gene had a lesser functional variation than might be expected; and positive scores pointed to variability exceeding the average level. Negative scores are therefore suggestive of purifying selection and positive scores of balanced or positive selection or both.

The statistical significance of differences between distributions was estimated by Fischer's *t*-test for angular transformed proportions (see above).

## 3. Results

### 3.1. Human Promoter Variability in the Whole-Genome Dataset

**Figure 1:**
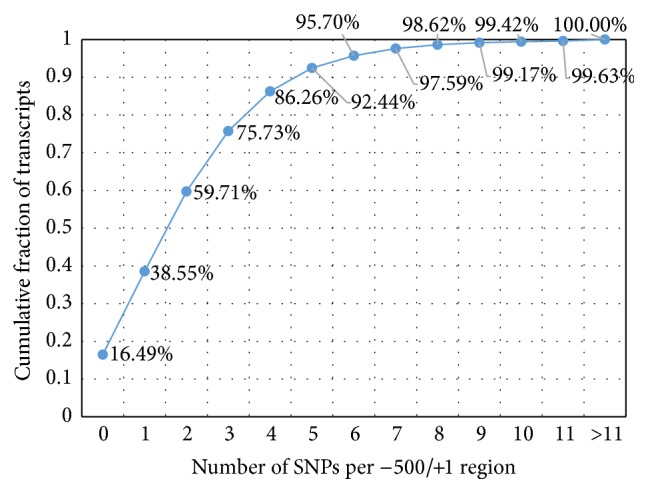
Cumulative percentage plot for the fraction of human transcripts from the whole-genome dataset, possessing certain numbers of SNPs in their 500 bp long regions upstream annotated transcription start sites. *x*-axis denotes the threshold SNP content in 500 bp upstream TSS. *y*-axis denotes the cumulative fraction of the whole-genome dataset of transcripts.

Figure 1 shows the fractions of human transcripts (from the whole-genome dataset of 47,469 protein-coding transcripts; see Section 2.1), possessing no more than certain numbers of SNPs (SNP content) in 500 bp long regions upstream annotated TSSs. We designated such a number of SNPs as a threshold for SNP content in the upstream region.

The majority of transcripts have low or intermediate SNP contents in their 500 bp regions upstream annotated TSSs. For example, no more than five SNPs were found in the upstream regions of 92.44% of transcripts. This means that the other transcripts of the whole-genome dataset (~8%) contain six or more SNPs in their 500 bp long upstream regions. Functional annotation of this group of transcripts with SNP-rich promoters (six or more SNPs per 500 bp) was presented previously [14]. At most one SNP was found in the upstream regions of 38.5% of transcripts. Almost one-sixth of transcripts from the whole-genome dataset (16.5%) do not contain SNPs in their 500 bp long upstream regions.

### 3.2. Proportions of Transcripts from the Whole-Genome Dataset That Do Not Contain SNPs in Their 5′-Regulatory Regions

To define the optimal length and location of the 5′-regulatory regions of genes that subsequently would be subjected to the functional analysis, we calculated the proportions of transcripts from the whole-genome dataset that do not contain SNPs in various bins of 5′-regulatory regions.

**Figure 2:**
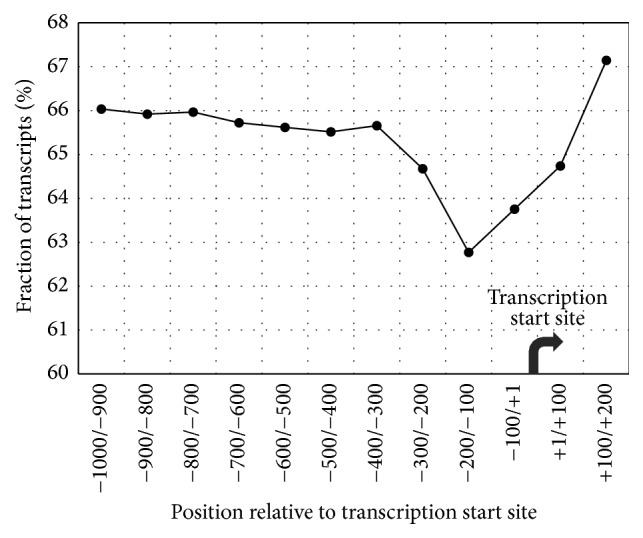
The fractions of transcripts from the whole-genome dataset that do not contain SNPs in their 100 bp long upstream regions as a function of region location. *x*-axis shows the borders of 100 bp bins relative to the TSS. *y*-axis means the fraction of transcripts that lack SNPs in the respective 100 bp bin.

Using data on SNPs content in each 100 bp bin from −1000 to +200, the proportions of transcripts in the whole-genome dataset that do not contain SNPs in a fixed bin were calculated (Figure 2). The minimal fraction of transcripts that lack SNPs in a 100 bp long bin was revealed for the local region from −200 to −100. Five local 100 bp long bins upstream (from −700 to −200) and two downstream bins (from −100 to +100) are also characterized by lower proportions of transcripts that lack SNPs within these bins. The bins at both flanks [−1000/−700] and [+100/+200] have greater proportions of transcripts that lack SNPs within these bins.

This analysis allows us to conclude that (1) the region [−300; +100] around TSSs has the highest content of SNPs; and (2) for subsequent analysis, the optimal 3′-boundary of promoter regions possessing elevated SNPs content may be strictly defined as +100 and the 5′-boundary of these regions may be set within a wide range from −700 to −300.

### 3.3. Biological Processes Overrepresented among Genes Whose Transcripts Were Found in the SNP-Depleted Datasets

To reveal the functional characteristics of genes whose promoter regions have low levels of polymorphisms, we performed functional analysis of genes whose transcripts had no SNPs in their 5′-flanking regions. The analysis was done for several datasets of transcripts that had no SNPs within extended or restricted 5′-regulatory regions (Table 2). In what follows, these datasets will be designated as* SNP-depleted within [*−*900/+100]*,* SNP-depleted within [*−*800/+100]*, and so forth until* SNP-depleted within [*−*300/+100]*. The numbers of SNP-depleted transcripts/genes involved into analysis are indicated in the second column of Table 2. The largest number of transcripts/genes was 10,488/6,024 for dataset* SNP-depleted within [*−*300/+100]* and the lowest number of transcripts/genes involved into analysis was 2,821/1,587 for dataset* SNP-depleted within [*−*900/+100].*

The GO terms overrepresented among transcripts/genes from* SNP-depleted datasets* were selected by applying the DAVID tool. The full list of the most overrepresented GO categories for each dataset is presented in Table S2. In all cases, fold enrichment exceeded 1.5, and *p* value was below 0.001. Then we composed a joint list of overrepresented GO terms revealed from analysis of all* SNP-depleted datasets* for various overlapping regions, removed duplicates, and grouped closely related GO terms (see columns 1 and 2 in Table S3). The next columns of this table summarize the occurrence of each GO term among the overrepresented terms for each* SNP-depleted dataset*. In such a way, we revealed three most common classes among GO terms satisfying the aforementioned criteria that were associated with (a) chromatin organization, (b) transcription, and (c) multicellular organism reproduction/gamete generation. GO terms that belong to these three classes are given in Table 2.

The first class of GO terms includes* regulation of specific transcription from RNA polymerase II promoter, positive regulation of specific transcription from RNA polymerase II promoter, negative regulation of transcription, DNA-dependent, regulation of gene-specific transcription, negative regulation of gene-specific transcription*, and* negative regulation of specific transcription from RNA polymerase II promoter*. The second class of GO terms includes* chromosome organization, chromatin organization, chromatin modification, chromatin assembly or disassembly, nucleosome organization*, and* DNA packaging*. The third class of GO terms includes* gamete generation, male gamete generation,* and* spermatogenesis*.

The numbers of genes annotated by GO categories of these three classes varied from 15 to 105. GO terms associated with* chromatin organization* and* transcription* were found to be overrepresented in four or more* SNP-depleted datasets*. GO terms associated with* transcription* were revealed on the base of analysis of SNP-depleted 5′-regulatory regions of short and medium lengths (−300/+100, −400/+100, −500/+100, and −600/+100) (Table 2). GO terms associated with* chromatin organization* were revealed on the base of analysis of SNP-depleted 5′-regulatory regions of short (−400/+100), medium (−500/+100 and −600/+100), and long lengths (−700/+100, −800/+100, and −900/+100) (Table 2). GO terms associated with multicellular organism reproduction/gamete generation were overrepresented in three* SNP-depleted datasets* formed based on the most extended regions (−700/+100, −800/+100, and −900/+100). For the latter three regions we found among top-scoring GO terms only terms associated with* chromatin organization* and* gamete generation* (Table S3).

As shown in Tables S2 and S3, the distinctive feature of the dataset* SNP-depleted within [*−*600/+100]* is that practically all (with only one exception) overrepresented GO terms (among those revealed with fold enrichment > 1.5 and *p* value < 0.001) are associated with* chromatin organization* and* transcription.* Hence, we performed the next analysis only for region −600/+100.

### 3.4. Promoter Variability in Genes Controlling Transcription and Chromatin Organization

Our second analysis was undertaken to compare promoter variability in genes controlling transcription or chromatin organization with the variability in the whole-genome dataset. Since genes encoding transcription factors are the largest functional group of genes associated with transcription, we included this gene group into analysis. Likewise, we chose genes encoding chromatin-modifying proteins for further functional analysis as genes functionally associated with chromatin organization. The lists of transcripts/genes encoding transcription factors or chromatin-modifying proteins were formed as described in Materials and Methods. These lists are denoted in what follows as* All TFs* and* Chr_Mod.*

To characterize the group of genes controlling transcription factors in more detail, we divided this list of genes into four subclasses according to the structures of their DBDs (see Section 2) and performed the same analysis with four sets of 5′-regulatory regions of genes from these four subclasses.

At this step of our analysis, only 5′-regulatory regions spanning nucleotides within −600 to +100 around TSSs were investigated.

**Figure 3:**
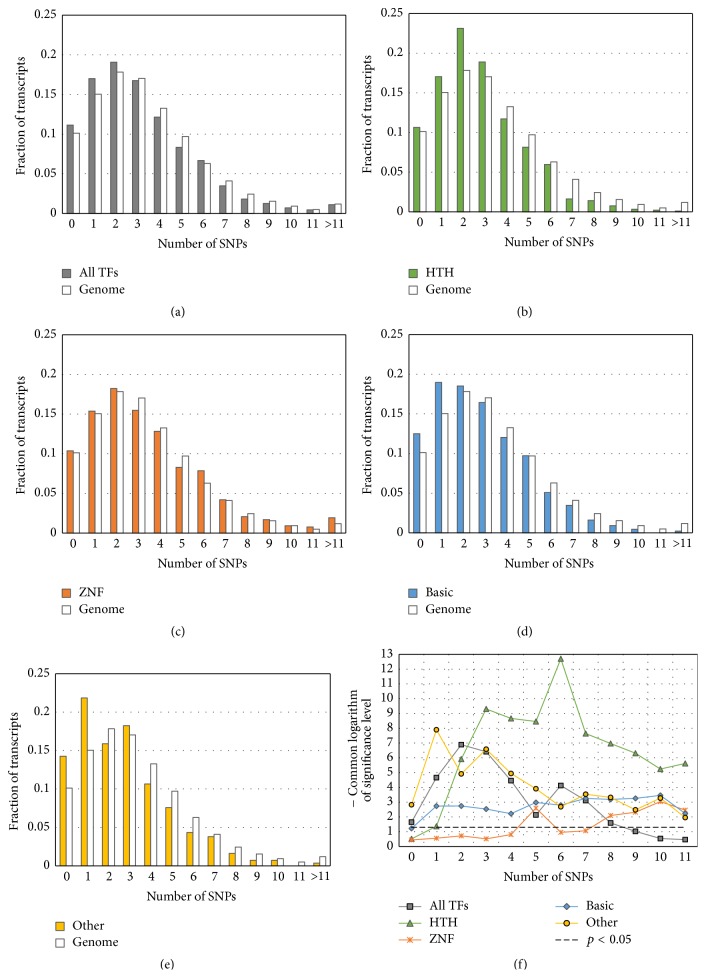
Comparison of SNP content distributions in upstream regions for human genes encoding transcription factors with the distribution in the whole-genome dataset (Table 1). The 5′-regulatory regions between −600 and +100 bp around TSSs are analyzed. The datasets of transcripts/genes (Table 1) are derived from TFClass [9]: (a) all transcription factors, (b) helix-turn-helix factor genes, (c) transcription factors with zinc-coordinating DBDs, (d) basic domain factor genes, and (e) genes encoding factors with DBDs of all other types. In panels (a) to (e) the *x*-axis denotes the SNP content; the *y*-axis means the fraction of transcripts with specific content of SNPs in their 5′-regulatory regions. Panel (f) presents the significance of the *t*-test (*y*-axis), which compare the above-described SNP contents in test groups with the content in the whole-genome dataset as a function of the threshold of SNP content (*x*-axis). The *t*-test was applied as described in Section 2.

**Figure 4:**
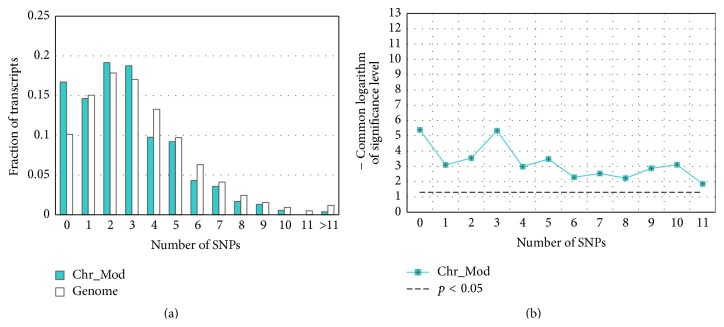
Comparison of SNP content distributions in upstream regions of human genes encoding chromatin-modifying proteins with the distribution in the whole-genome dataset (Table 1). The 5′-regulatory regions between −600 and +100 bp around TSSs are analyzed. The dataset of chromatin-modifying proteins genes/transcripts was extracted from EntrezGene, CREMOFAC [10], and CR Cistrome Databases [11]. In panel (a), the *x*-axis denotes the SNP content, and the *y*-axis denotes the fraction of transcripts with specific content of SNPs in their 5′-regulatory regions. Panel (b) presents the significance of the *t*-test (*y*-axis), which compares the SNP contents in test group with the content in the whole-genome dataset as a function of the threshold of SNP content (*x*-axis). The *t*-test was applied as described in Section 2.

The comparison of distributions of SNP content in 700 bp long regions (from −600 to +100) around annotated TSSs in each group of transcripts and in the whole-genome dataset shows that transcripts of both large groups (*All TFs* and* Chr_Mod)* and three subclasses of TFs (groups* HTH*,* Basic*, and* Other*) tend to have lower SNP contents (Figures 3(a), 3(b), 3(d), 3(e), and 4(a)). To confirm this assumption, we applied the *t*-test for angular transformed proportions (see Materials and Methods) to the range of thresholds of SNP content (Figures 3(f) and 4(b)). We concluded that for any threshold of SNP content from one to eight significant depletion of transcripts with SNPs was observed in five out of six gene groups. A very weak significance at only one threshold (“≤5”) was revealed for the group that comprised genes encoding factors with zinc-coordinating DBDs (Figures 3(c) and 3(f)).

### 3.5. Promoter Variability in Genes Controlling Spermatogenesis

To interpret the low SNP content in the 5′-regulatory regions of genes controlling gamete generation, we additionally compiled the dataset of genes* Sperm* (Table 1).

**Figure 5:**
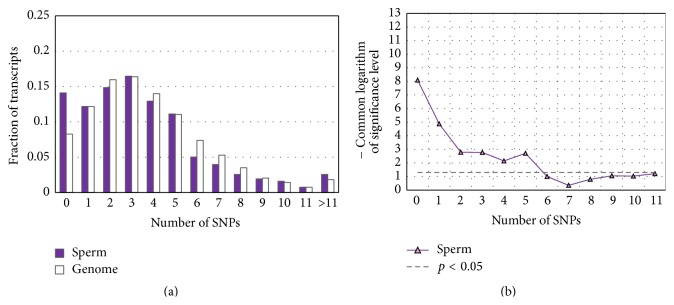
Comparison of SNP content distributions in the upstream regions of human genes controlling spermatogenesis (dataset* Sperm*) with the distribution in the whole-genome dataset (Table 1). The 5′-regulatory regions between −700 and +100 bp around TSSs were analyzed. The genes for dataset* Sperm* were extracted from EntrezGene by GO term* spermatogenesis*. In panel (a), the *x*-axis denotes the SNP content, and the *y* axis denotes the fraction of transcripts with specific contents of SNPs in their 5′-regulatory regions. Panel (b) presents the significance of the *t*-test (*y*-axis), which compares the SNP contents in test group with the content in the whole-genome dataset as a function of the threshold of SNP content (*x*-axis). The *t*-test was applied as described in Section 2.

At this step of our analysis, 5′-regulatory regions spanning nucleotides within −700 to +100 around TSSs were investigated. This region was chosen in accordance with the fact that the GO terms associated with spermatogenesis were enriched only in datasets* SNP-depleted within [*−*700/+100], SNP-depleted within [*−*800/+100], and SNP-depleted within [*−*900/+100]* (Tables 2, S2, and S3). The comparison of distribution of the SNP content in 5′-regulatory regions (from −700 to +100) in a group of transcripts denoted as* Sperm* and in the whole-genome dataset shows that transcripts of this functional group tend to have lower SNP contents (Figure 5(a)). The *t*-test (Figure 5(b)) confirms that the differences are significant.

However, we found that a notable portion of transcripts from the* Sperm* dataset (52 transcripts out of 936) were located on the Y chromosome, while only 115 transcripts out of the total amount 47,469 were mapped to this chromosome (according to *t*-test *p* < 2*∗*10^−30^). We suspected that this notable enrichment might explain the extremely low level of SNPs in 5′-regulatory regions of transcripts from the* Sperm* dataset, since there were profound differences between the SNP content in 5′-regulatory gene regions located on chromosomes 1–22 and the SNP contents on both sex chromosomes (Figure S1).

To test this hypothesis, we excluded transcripts located on the X and Y chromosomes from all datasets presented in Table 1 and performed the same analysis as in the previous section. The results are presented in Figures S2(a), S2(b), and S2(c).

We revealed that, only for genes from the dataset* Sperm*, (1) the difference in promoter SNP content between transcripts located on all chromosomes (autosomes and sex chromosomes) and the whole-genome dataset of transcripts was significant (Figure 5), while (2) for the respective pair of autosomal subsets of transcripts (spermatogenesis versus whole-genome) the statistical significance was rejected (Figure S2(c)). Hence, the depletion of SNPs in the 5′-regulatory regions of spermatogenesis genes is the consequence of the frequent occurrence of these genes in the Y chromosome. For this reason, hereafter we consider only genes controlling transcription and chromatin organization.

### 3.6. Estimations of Genic Intolerance to Functional Variation in Functional Gene Groups and Comparison with the Whole-Genome Dataset

Since low levels of SNPs in the 5′-regulatory regions of genes encoding TFs and chromatin-modifying proteins may reflect the selection pressure acting on genes, the question arises as to the extent to which the promoter SNP content correlates with other measures of selection pressure on genes. For this purpose, we used RVIS values, which had been calculated by Petrovski et al. [21], based on data on allele frequencies in the coding gene regions of genes (for details see Section 2). RVIS ranks human genes in terms of their intolerance to permanent functional genetic variation in the human population.

**Figure 6:**
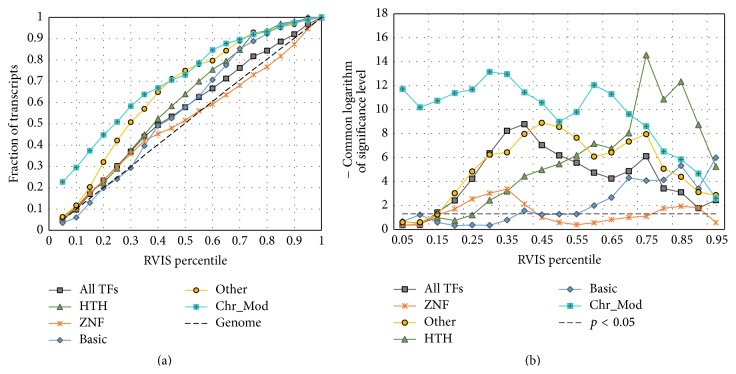
Comparison of Residual Variation Intolerance Score (RVIS) [21] percentiles for six groups of human genes with percentiles for the whole-genome dataset. (a) Cumulative percentage plots for the RVIS percentiles for six groups of human genes and for the whole-genome dataset. (b) Significance of the *t*-test (*y*-axis) where RVIS percentiles in six groups were compared with those for the whole-genome dataset as a function of the RVIS percentile threshold (*x*-axis). The *t*-test was applied as described in Section 2.

We found that genes encoding TFs and chromatin-modifying proteins had lower RVIS values than genes from the whole-genome dataset (Figure 6(a)). The comparison of RVIS values for subclasses of TFs with different types of DBDs revealed that (a) group* Other*, which comprised 160 TFs with DBDs of seven different types, was the most intolerant to genetic variation and (b) the group of genes encoding factors with zinc-coordinating DBDs was the most tolerant to functional genetic variation.

The application of the *t*-test (see Section 2) for distributions of SNP content showed that the differences between datasets* All TFs*,* Chr_Mod*, and three out of four subclasses of genes encoding TFs (*HTH, Basic, *and* Other*) versus genes from the whole-genome dataset were highly significant for the broad range of thresholds (Figure 6(b)).

Thus, the subclasses of TFs can be ranked according to RVIS with regard to intolerance to mutations in their coding regions as follows:* Other* >* HTH* ~* Basic* >* ZNF*. This order perfectly correlates with the differences between these gene groups and the whole-genome dataset revealed when distributions of promoter SNP content were analyzed (Figure 3(f)).

## 4. Discussion

### 4.1. Fractions of Transcripts with Elevated or Reduced SNP Contents in the 5′-Regulatory Regions of Human Protein-Coding Genes

At the first step of our study, we confirmed our previous result [14] that SNP contents in the 5′-flanking regions of protein-coding genes from the whole-genome dataset were highly variable (Figure 1). We used 1000 Genomes Project data from dbSNP build 142 and annotation of transcripts extracted from the Ensembl.

Almost one-twelfth (7.5%) of the total number of promoters were found to have high SNP contents (six or more SNPs). This is in good agreement with our earlier report based on data from dbSNP build 138 [14], where we showed that genes with greater genetic variability of their 5′-flanking regions (more than six SNPs per 500 bp) comprised 5.5% of all human genes. According to functional annotation performed in that study by DAVID tool, three groups were overrepresented among the genes with high SNP content: (a) genes controlling the sensory perception of smell, (b) a specific subset of promoters of sensory perception genes encoding olfactory receptors, and (c) genes involved in antigen processing and presentation. It was proposed that the elevated level of genetic variability in promoter regions of these functional groups of genes is maintained to an extent by balancing selection, that is, the necessity of evolutionary adaptation to highly variable environmental conditions characterized by great diversity of immunogenic and olfactory stimuli.

On the other hand, one-fifth (16.5%) of the total number of 5′-regulatory regions within −500/−1 were SNP-depleted. We were also interested in investigating the extent to which the proportions of SNP-depleted promoters depend on the length and location of the promoter regions. The analysis of proportions of transcripts having no SNPs within particular 100 bp bins in the −1000/+200 regions around TSSs showed that (a) the bins between −200 and −100 had the lowest proportion and (b) bins within −700 and +100 had lower proportions than flanking ones (Figure 2).

The reduced proportion of SNP-depleted regions among local 100 bp regions within −300/+100 revealed in our study tells us that these regions surrounding TSSs contain elevated numbers of SNPs in comparison with their flanking regions.

This finding agrees with the results published previously by [25, 26]. Both studies had shown that in 5′-regions of human genes more SNPs occurred in close proximity to transcriptional start sites (200–300 bp in length) than in regions further upstream. Moreover, according to [26], SNPs were more abundant in the first 100 nucleotides downstream TSS than in other downstream regions.

Taking in account our results (Figure 2) and previously published data, we conclude that the −700/+100 regions are interesting for further functional analysis. To obtain a more detailed view and to be sure that we did not miss any important detail, at the next step of our study we performed functional analysis based on data calculated for a more wider range of 5′-flanking regions (from [−900; +100] and [−800; +100] to [−400; +100] and [−300; +100]).

### 4.2. Reduced Genetic Variability in the Promoter Regions of Genes Encoding Transcription Factors and Chromatin-Modifying Proteins

Functional analysis of SNP-depleted transcripts performed with the DAVID tool revealed three distinct classes of overrepresented GO terms (Table 2). The first class was associated with* transcription regulation*. The second consisted of GO terms related to* chromatin organization*. Notably, these two classes define two extremely important biological processes, transcription regulation by transcription factors, and regulation of chromatin packaging. Both biological processes were overrepresented in four or more sets of SNP-depleted transcripts, indicating that this finding was highly reliable. The third class, smaller than the two, listed terms that described a highly tissue-specific process of male gamete generation (spermatogenesis). We considered this class of GO terms because terms related to male gamete generation were found for* SNP-depleted datasets* of transcripts with very long 5′-regions ([−900; +100], [−800; +100], and [−700; +100]). Such extended lengths of 5′-regions might point to significance of the finding. However, the statistical *t*-test on the heterogeneity of SNP content among the 5′-regulatory regions of genes mapped to autosomes or sex chromosomes (Figure S2) showed that the low level of SNPs in the third class might be completely explained by more frequent (than expected) location of genes involved in spermatogenesis on the Y chromosome. Hence, we restricted our subsequent analysis of genetic variability within 5′-regulatory regions to two groups of genes involved in* transcription* and* chromatin organization* (corresponding to the two major classes of GO terms).

By using public databases, we created lists of genes encoding TFs and chromatin-modifying proteins (Tables 1 and S1). Afterwards, we compared the contents of SNPs in the 5′-regulatory regions of transcripts (−600/+100) of the aforementioned groups (*All TFs* and* Chr_Mod*) with that for the whole-genome dataset by Fischer's *t*-test for angular transformed proportions. We showed that 5′-regulatory regions of both groups had reduced genetic variability in comparison to that in the whole-genome dataset (Figures 3(f) and 4(b)).

### 4.3. Functional Similarity and Parallelism between Transcription Factors and Chromatin-Modifying Proteins

The whole-genome analysis of the SNP content in 5′-regulatory regions revealed two interesting groups of genes with reduced genetic variability: genes encoding transcription factors and genes encoding chromatin-modifying proteins. The biological functions of these two groups of genes are closely similar.

Sequence-specific DNA-binding TFs direct transcription initiation to specific promoters through binding to certain* cis*-regulatory elements in promoters, enhancers, silencers, and other regulatory regions [2, 27]. The effects of their binding may be (a) facilitation of the formation of the basal transcription complex through contacts to general transcription factors or (b) triggering of chromatin remodeling through DNA or histone modifications [8].

Genome-wide measurements of protein-DNA interactions combined with analysis of gene expression profiles have shown that each transcription factor can modulate transcription levels of thousands of target genes adjusting activities of genes within gene networks [28, 29].

Chromatin-modifying proteins can (a) posttranslationally modify and demodify chromatin, altering chromatin structure and recruiting regulatory factors and (b) provide access to nucleosomal DNA or allow nucleosomes to move to a different position along the DNA, remove, or exchange nucleosomes using energy from ATP hydrolysis [11]. Genome-wide analysis of histone modifications revealed that, like transcription factors, each chromatin-remodeling protein can affect transcriptional level of thousands of genes, thereby orchestrating gene activity according to intracellular conditions or external stimuli [30].

Thus, both classes of proteins are involved in the complicated process of transcriptional control, ensuring correct expression of specific genes. Both so called “transcription factor-binding regulatory code” and “histone code” may be effectively used for prediction of gene expression activity. Moreover, these codes are redundant for predicting gene expression [31]. This redundancy means that TFs and chromatin-modifying proteins function in close cooperation, facilitating the recruitment of each other to transcription complexes. The numerous protein-protein interactions revealed between TFs and chromatin-modifying proteins convincingly prove this idea [32, 33].

The transcriptional regulatory system plays the central role in controlling many biological processes, ranging from cell cycle progression and maintenance of intracellular metabolic and physiological balance, to cell differentiation and developmental time courses. Numerous diseases arise from a breakdown in the regulatory system: transcription factors are overrepresented among oncogenes [34], and a third of human developmental disorders are attributed to dysfunctional TFs [27, 35, 36]. For example, genes encoding transcriptional regulators constitute a substantial proportion of genes associated with autism [37]. Disruption in the activity of gene expression regulators, such as transcription factors and chromatin-remodeling proteins, accounts for the expression changes observed in multiple animal and cellular models of Huntington's disease and in samples from patients [38].

Therefore, it is not surprising that genes of both these systems have lower SNP contents in their 5′-regulatory regions. So we hypothesize that the reduced variability of regulatory regions may be due to selective pressure (purifying selection), which removes deleterious alleles. To estimate the force of selective pressure on genes encoding TFs and chromatin-modifying proteins, we calculated RVIS values. According to cumulative percentage plots for the RVIS percentiles (Figure 6(a)), both groups showed reduced intolerance to functional variation, confirming our hypothesis that a low level of promoter polymorphism may have resulted from purifying selection.

### 4.4. The Differences between Superclasses of TFs

According to Figure 3(a), TFs have reduced SNP contents in their 5′-regulatory regions. In addition, we found sharp differences between four sets of transcripts/genes encoding TFs with DBDs of specific types. For two groups of genes encoding TFs (TFs with helix-turn-helix DBDs (HTH superclass) and TFs with DBDs of seven other superclasses (with the exception of the largest ones* ZNF, HTH,* and* Basic*; see Section 2)), the differences against the whole-genome dataset were highly significant in a wide range of SNP contents in the 5′-regulatory regions (Figures 3(b), 3(e), and 3(f), groups* HTH* and* Other*). The group of genes encoding TFs with the basic domain demonstrated a moderate significance level (Figures 3(d) and 3(f), group* Basic*). Finally, practically no differences were revealed between genes encoding TFs with zinc-coordinating DBDs and the whole-genome dataset (Figures 3(c) and 3(f), group* ZNF*). The differences between groups of genes formed according to the types of encoding proteins were reproduced when cumulative percentage plots for the RVIS percentiles were built (Figure 6). Thus, according to RVIS, genes encoding TFs with DBDs of seven other types are the most intolerant to functional variation.

According to [21], low RVIS points to high functional significance of the gene and predicts potential association with diseases. Indeed, among this group of TFs with DBDs of seven other types (denoted as* Other*) there are many well-known hubs in a molecular network. They include (a) TP53 involved in cell cycle control [39, 40]; (b) TBPs that provide the recognition of the TATA box within the core promoter [41, 42]; (d) factors from the NF-kB (NFKB1, NFKB2, NFKBIA, NFKBIB, NFKBID, NFKBIE, NFKBIL1, and NFKBIZ) and STAT (STAT1, STAT2, STAT3, STAT4, STAT5A, STAT5B, and STAT6) families participating in immune response [43–46]; and (e) factors from the SOX family (SOX2, SOX5, SOX7, SOX12, and SOX15), which regulate the network of genes that orchestrate mammalian embryogenesis [47, 48].

On the other hand, the group of genes encoding transcription factors with zinc-coordinating DBDs (*ZNF*) was a special case, because it did not differ from the whole-genome dataset (Figure 3, panels (c) and (f)). This observation was in agreement with data obtained for RVIS values (Figure 6). A very weak enrichment of this group of TFs in comparison with the whole-genome dataset was revealed between 20th and 40th percentiles of RVIS values.

The distinctive features of TFs with zinc-coordinating DBDs revealed in our study are in accordance with ideas proposed in [49]. They presented an evolutionary analysis of poly-zinc-finger gene family and showed that zinc-finger genes were not conserved among mammals. Zinc-finger genes have undergone extensive expansion in humans. The human genome encodes approximately 700 members of this superclass. It was demonstrated that the major component of the selective pressure acting on these genes was positive selection to change their DNA-binding specificity. We suggest that in humans, owing to the large number of genes comprising the zinc-finger gene family, many of them encode proteins with very similar functions. That is why some alleles in promoter regions controlling zinc-finger genes at least temporarily escape eradication by purifying selection.

The second circumstance that could partly explain distinctive features of TFs with zinc-coordinating DBDs was outlined in the paper devoted to TFClass [8]. This superclass was characterized by an elevated (in comparison with other superclasses) content of putative transcription factors. This means that for a substantial proportion of zinc-finger genes the functional roles of encoded proteins were not studied experimentally.

## 5. Conclusions

This study demonstrates that the genes involved in gene-specific transcription (especially regulatory sequence-specific DNA-binding factors) and chromatin organization (especially chromatin-modifying proteins) are overrepresented among genes whose promoters do not contain SNPs. This observation points to a lower tolerance of these groups of genes to regulatory genetic variation. Our finding may give a deeper view of genic intolerance to regulatory variation and may be useful for interpretation of personal genomes.

## Supplementary Material

Figure S1: Human promoter variability in the whole-genome dataset (Table 1) compared with promoter variability in datasets from chromosomes 1-22 (autosomes) or chromosome X, or chromosome Y. Cumulative percentage plots for the fractions of human transcripts, possessing certain numbers of SNPs in [–600/+100] regions relative to annotated transcription start sites are presented.
Figure S2: The comparison of SNP content distributions in upstream regions of seven groups of human transcripts (All TFs, ZNF, HTH, Basic, Other, Chr Mod, Sperm) from chromosomes 1-22 (transcripts from the chromosomes X and Y were excluded from analysis) with the distribution in the whole-genome dataset from which transcripts located on the chromosomes X and Y were also excluded.
The significances of the t test (Y axis), which compares SNP contents in seven groups of human transcripts with the content in the whole genome dataset as a function of the SNP content (X axis) are presented. Panel A: All TFs, ZNF, HTH, Basic, Other; Panel B: Chr Mod; Panel C: Sperm.
Table S1:Datasets of transcripts, described in the Table 1: All TFs, ZNF, HTH, Basic, Other, Chr Mod, Sperm.
HGNC Gene Name, Ensembl Gene ID, Ensembl, Transcript ID, SNP count are presented.
Table S2:The full list of the most overrepresented (fold enrichment > 1.5 and *p* value < 10-3) GO categories for each SNP-depleted dataset.
Table S3: The joint list of overrepresented (fold enrichment > 1.5 and *p* value < 10-3) GO terms revealed from analysis of all SNP-depleted datasets (The duplicates of GO terms were removed).
Columns from C to I present the occurrence of each GO term among the overrepresented terms for each SNP-depleted dataset.

## Figures and Tables

**Table 1:** Sequence sets used in analysis. The column “number of transcripts/genes” presents the count of transcripts that were computed after intersection of the respective subset with the whole-genome dataset of 47,469/18,817 transcripts/genes.

Dataset of transcripts		Number of transcripts/genes
Full name	Short name	
All protein-coding transcripts from the human genome	*Whole-genome*	47,469/18,817

All transcription factors from TFClass	*All TFs*	3,957/1,454

Transcription factors with zinc-coordinating DBDs (Superclass 2 from TFClass)	*ZNF*	2,074/750

Helix-turn-helix transcription factor genes (Superclass 3 from TFClass)	*HTH*	921/383

Basic domain transcription factor genes (Superclass 1 from TFClass)	*Basic*	432/170

Genes encoding transcription factors with DBDs of all other types (Superclasses 0, 4, 5, 6, 7, 8, and 9 from TFClass)	*Other*	554/160

Genes encoding chromatin-modifying proteins (according to GO, CREMOFAC, and CR Cistrome)	*Chr_Mod*	533/167

Genes encoding proteins involved in spermatogenesis (according to GO)	*Sperm*	936/361

**Table 2:** Gene ontology (GO) terms significantly overrepresented (*p* < 0.001, fold enrichment > 1.5) in the *SNP-depleted* datasets of human transcripts. Each dataset comprises transcripts that have no SNPs within specific 5′-regulatory regions relative to TSS (first column). The list of overrepresented GO terms was generated by the DAVID tool [22, 23]. Designations of GO terms: italicization means association with *transcription*, boldface denotes *chromatin* or *chromosome organization*, and the regular  font points to *spermatogenesis* and *gamete generation*.

Location	The total number of transcripts/genes	GO term name	Number of genes involved in the term	*p* value	Fold enrichment
−300/+100	10,488/6,024	*Regulation of specific transcription from RNA polymerase II promoter*	47	8.2*E* − 05	1.67
		*Positive regulation of specific transcription from RNA polymerase II promoter*	30	7.4*E* − 04	1.76

−400/+100	7,799/4,572	**Chromatin modification**	93	1.4*E* − 05	1.51
		*Regulation of specific transcription from RNA polymerase II promoter*	38	1.7*E* − 04	1.80
		*Positive regulation of specific transcription from RNA polymerase II promoter*	25	7.2*E* − 04	1.95

−500/+100	6,096/3,572	**Chromatin organization**	105	3.7*E* − 07	1.60
		*Negative regulation of transcription, DNA-dependent*	99	7.8*E* − 07	1.61
		**Chromatin modification**	77	9.8*E* − 06	1.62
		*Regulation of specific transcription from RNA polymerase II promoter*	32	1.6*E* − 04	1.97
		*Regulation of gene-specific transcription*	41	2.3*E* − 04	1.77
		*Negative regulation of transcription from RNA polymerase II promoter*	70	2.3*E* − 04	1.52

−600/+100	4,805/2,786	**Chromatin organization**	89	4.7*E* − 08	1.78
		**Chromosome organization**	99	8.3*E* − 06	1.54
		*Regulation of specific transcription from RNA polymerase II promoter*	29	2.0*E* − 05	2.33
		*Negative regulation of transcription, DNA-dependent*	76	2.2*E* − 05	1.61
		**Chromatin modification**	62	2.3*E* − 05	1.71
		*Negative regulation of transcription from RNA polymerase II promoter*	57	2.4*E* − 04	1.62
		*Regulation of gene-specific transcription*	34	2.4*E* − 04	1.92
		*Negative regulation of gene-specific transcription*	17	2.8*E* − 04	2.68
		*Negative regulation of specific transcription from RNA polymerase II promoter*	15	5.1*E* − 04	2.77
		**Chromatin assembly or disassembly**	31	9.6*E* − 04	1.85

−700/+100	3,926/2,252	**Chromatin organization**	75	6.7*E* − 08	1.89
		**Chromosome organization**	83	7.2*E* − 06	1.63
		**Chromatin modification**	51	6.2*E* − 05	1.78
		Spermatogenesis	53	3.4*E* − 04	1.64
		Male gamete generation	53	3.4*E* − 04	1.64
		**Nucleosome organization**	22	5.1*E* − 04	2.26
		Gamete generation	63	7.3*E* − 04	1.52
		**DNA packaging**	25	9.2*E* − 04	2.04

−800/+100	3,287/1,858	**Chromatin organization**	66	1.4*E* − 08	2.09
		**Chromosome organization**	72	2.0*E* − 06	1.78
		**Chromatin modification**	44	4.0*E* − 05	1.92
		Spermatogenesis	47	7.6*E* − 05	1.83
		Male gamete generation	47	7.6*E* − 05	1.83
		**Nucleosome organization**	19	6.0*E* − 04	2.45
		Gamete generation	53	6.6*E* − 04	1.61
		**Chromatin assembly or disassembly**	23	7.8*E* − 04	2.17

−900/+100	2,821/1,587	**Chromatin organization**	62	7.2*E* − 10	2.33
		**Chromosome organization**	68	6.8*E* − 08	1.99
		**Chromatin modification**	41	8.2*E* − 06	2.12
		Male gamete generation	42	5.9*E* − 05	1.94
		Spermatogenesis	42	5.9*E* − 05	1.94
		**Nucleosome organization**	19	7.2*E* − 05	2.90
		**Chromatin assembly or disassembly**	22	2.0*E* − 04	2.46
		**DNA packaging**	20	5.0*E* − 04	2.43
		Gamete generation	47	5.0*E* − 04	1.69
