# Supplementary material for: Human Genes Encoding Transcription Factors and Chromatin-Modifying Proteins Have Low Levels of Promoter Polymorphism: A Study of 1000 Genomes Project Data
Source: Int J Genomics. 2015 Aug 31;2015:260159. doi: 10.1155/2015/260159 (PMC4568383; doi:10.1155/2015/260159)
Supplement: Supplementary file 1 — Figure S1: Human promoter variability in the whole-genome dataset (Table 1) compared with promoter variability in datasets from chromosomes 1-22 (autosomes) or chromosome X, or chromosome Y. Cumulative percentage plots for the fractions of human transcripts, possessing certain numbers of SNPs in [–600/+100] regions relative to annotated transcription start sites are presented. Figure S2: The comparison of SNP content distributions in upstream regions of seven groups of human transcripts (All TFs, ZNF, HTH, Basic, Other, Chr Mod, Sperm) from chromosomes 1-22 (transcripts from the chromosomes X and Y were excluded from analysis) with the distribution in the whole-genome dataset from which transcripts located on the chromosomes X and Y were also excluded. The significances of the t test (Y axis), which compares SNP contents in seven groups of human transcripts with the content in the whole genome dataset as a function of the SNP content (X axis) are presented. Panel A: All TFs, ZNF, HTH, Basic, Other; Panel B: Chr Mod; Panel C: Sperm. Table S1:Datasets of transcripts, described in the Table 1: All TFs, ZNF, HTH, Basic, Other, Chr Mod, Sperm. HGNC Gene Name, Ensembl Gene ID, Ensembl, Transcript ID, SNP count are presented. Table S2:The full list of the most overrepresented (fold enrichment > 1.5 and p value < 10-3) GO categories for each SNP-depleted dataset. Table S3: The joint list of overrepresented (fold enrichment > 1.5 and p value < 10-3) GO terms revealed from analysis of all SNP-depleted datasets (The duplicates of GO terms were removed). Columns from C to I present the occurrence of each GO term among the overrepresented terms for each SNP-depleted dataset. [file 260159.f1.zip › Suppl_Figs_S1_S2.doc]

**Part 1.** Human promoter variability in the whole-genome dataset (Table 1) compared with promoter variability in datasets from chromosomes 1-22 (autosomes) or chromosome X, or chromosome Y. Totally 45324/17970, 2030/799 and 115/48 transcripts/genes were mapped to autosomes, X and Y chromosomes, respectively.

**Figure S1**: Cumulative percentage plot for the fraction of human transcripts from the whole genome dataset, possessing certain numbers of SNPs in [-600/+100] regions relative to annotated transcription start sites. X axis, the SNP content in region [-600/+100]. Y axis, the cumulative fraction of the whole genome dataset of transcripts.

**Part 2. Comparison of SNP content distributions in upstream regions of groups of human transcripts from chromosomes 1-22 (transcripts from the chromosomes X and Y were excluded from analysis) with the distribution in the whole-genome dataset from which transcripts located on the chromosomes X and Y were excluded.**

**Figure S2:** The significances of the *t* test (Y axis), which compare the above-described SNP contents in seven groups (with the exception of transcripts, located on the chromosomes X and Y) with the content in the whole genome dataset (with the exception of transcripts, located on the chromosomes X and Y) as a function of the SNP content (X axis). The *t* test was applied as described in Materials and Methods. The location of the 5’-regulatory regions for analysis was selected according to occurrence of respective top-scoring GO terms in various regions (Table 2, Supplementary Table 1). Panels A, B: the region -600 / +100 around TSSs was taken for (a) *All TF* (All transcription factors) dataset and its subsets *HTH, ZNF, Basic* and *Other*; (b) *Chr_Mod* (chromatin-modifying proteins) dataset. Panel C: the region -700/+100 around TSSs was taken for *Sperm* (spermatogenesis) dataset.
